# An Ultrasonic Micro-Tool Assisted Platform for Post-Processing of Micrometer-Scale Copper Wires

**DOI:** 10.3390/mi17040411

**Published:** 2026-03-27

**Authors:** Xu Wang, Zhiwei Xu, Chengjia Zhu, Tian Zhang, Qiang Tang, Junchao Zhang, Yinlong Zhu

**Affiliations:** 1 College of Mechanical and Electronic Engineering, Nanjing Forestry University, Nanjing 210037, China; xuzwei@njfu.edu.cn (Z.X.); zhuchengjia@njfu.edu.cn (C.Z.); zhangtian202501@163.com (T.Z.); 2 Jiangsu Key Laboratory of Advanced Manufacturing Technology, Faculty of Mechanical and Material Engineering, Huaiyin Institute of Technology, Huaian 223003, China; tangqiang102@126.com; 3 College of Aeronautics and Astronautics, Nanjing University of Aeronautics and Astronautics, Nanjing 210016, China; 15565369735@163.com

**Keywords:** ultrasonic microactuation-assisted micro-forming, fine copper wire, micro-tool design, micro-tool actuation, precision micro-manufacturing

## Abstract

Acoustic microactuation technology has emerged as an effective approach for fabrication of micro- and nanoscale objects, enabling precise processing and shaping control of microscale materials by efficiently transmitting ultrasonic vibration energy and focusing energy locally. In this work, the proposed platform is regarded as an acoustically driven micromachine, in which ultrasonic excitation acts as the primary microactuation mechanism. Micrometer-scale copper wires are widely used in microelectronics and precision manufacturing. However, their small dimensions and low rigidity make fixation and forming particularly challenging. To achieve controllable forming of fine copper wires, this study introduces an ultrasonic vibration energy-focusing principle and investigates an ultrasonic post-processing method tailored for such materials, with the aim of enhancing processing stability and forming accuracy. An ultrasonic processing experimental platform for copper wires was established, and multiple micro-tool designs—including glass fiber, 304 stainless steel wire with support, and elastic hard 304 stainless steel—were evaluated. Single-point and continuous processing experiments were conducted by varying micro-tool materials and support configurations, and the influence of feed speed on processing width and depth was systematically analyzed. The results indicate that a hard 304 stainless steel micro-tool supported by a hard plastic ring provides the best overall performance. Feed speed has a significant effect on processing depth, with a maximum average depth of approximately 0.95 μm achieved at a feed speed of 1 mm/min. These findings demonstrate the feasibility of ultrasonic processing for the effective forming of fine copper wires and confirm that appropriate micro-tool design and feed speed are critical for achieving stable and reliable processing results. The proposed system employs an ultrasonically actuated micro-tool to perform post-processing on micrometer-scale copper wires. The ultrasonic vibration serves as a microactuation mechanism that enhances local deformation and material response during micro-machining.

## 1. Introduction

Microscale copper wires have broad application prospects in fields such as micro-interconnects, sensing elements, and flexible electronic devices, due to their excellent electrical conductivity, thermal conductivity, and good ductility [1]. With the continuous miniaturization of electronic systems, the demand for fine copper wires with diameters at the micrometer scale has increased significantly. However, as the wire diameter decreases, the mechanical rigidity and structural stability of copper wires are greatly reduced, making precise fixation and controllable forming extremely challenging during processing [2]. Conventional processing methods for metallic wires, such as mechanical rolling, drawing, and laser-based processing, face significant limitations when applied to fine copper wires. Mechanical methods often suffer from poor dimensional control and wire damage due to excessive contact forces, while laser processing may introduce thermal effects that degrade material properties at the microscale. Moreover, conventional mechanical processing usually results in burr formation and surface damage and provides limited dimensional accuracy, thereby failing to satisfy high-precision fabrication requirements [3].

Previous studies have demonstrated that ultrasonic vibration can effectively reduce forming forces, improve surface quality, and enable microscale material deformation. These advantages suggest that ultrasonic processing has strong potential for the forming of fine copper wires, particularly in applications requiring high precision and minimal thermal influence. Ultrasonic processing technology alters the interaction state at the tool–workpiece interface by superimposing high-frequency micro-vibrations, which can effectively reduce interface friction and machining forces, and improve processing stability and surface integrity [4]. In the field of micro metal machining, ultrasonic micro-machining (UMM) has been shown to enhance material removal efficiency and improve machining quality, particularly demonstrating significant advantages in the machining of high aspect ratio microstructures [5,6].

However, despite these advances, there is still a lack of systematic studies on how ultrasonic vibration characteristics influence the microstructural evolution, mechanical responses, and defect formation mechanisms of microscale copper wires during micro-forming or post-processing [7,8,9]. This limited understanding constrains the effective optimization of ultrasonic micro-machining for precision forming and interconnect fabrication of ultra-fine copper wires [10,11]. Moreover, beyond material-level responses, the influence of system-level factors, such as micro-tool (MT) structure, material stiffness, and support configuration, on ultrasonic energy transmission and forming stability has not been comprehensively investigated [12,13,14]. Quantitative investigations into the effects of processing parameters, including feed speed, on forming width and depth are also lacking. These unresolved issues collectively impede the systematic development and practical implementation of ultrasonic-assisted forming techniques for microscale wire processing [15,16].

Based on the limitations mentioned above, this study focuses on the mechanism by which ultrasonic vibration influences the micro-deformation behavior and surface integrity of micron-scale copper wires during ultrasonic micro-machining and post-processing [17,18]. Building on previous research and preliminary experimental observations, it is proposed that ultrasonic vibration may alter the material deformation mechanisms of micron-scale copper wires by alleviating local stress concentrations, promoting uniform plastic flow, and suppressing the initiation of surface defects [19,20,21]. To verify this inference, an ultrasonic-assisted micro-machining experimental system was constructed, and post-processing experiments on micron-scale copper wires were carried out on this platform. The experimental design involves varying ultrasonic parameters and the micro-tool while monitoring the post-processed surface morphology, dimensional accuracy, and microstructural evolution. Quantitative evaluation and comparative analysis of the machining results were conducted using optical microscopy and surface profile measurements, in order to reveal the effects of ultrasonic vibration of proposed structures on the forming behavior and surface integrity of fine copper wires.

## 2. Experimental Setup and Working Mechanism

### 2.1. Ultrasonic Micro-Processing System Design

**Figure 1:**
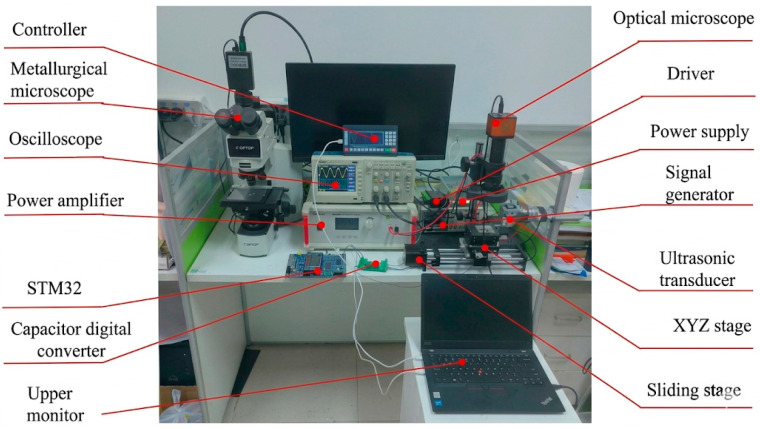
Experimental platform device diagram.

As shown in Figure 1, the ultrasonic micro-processing system integrates a signal generator, power amplifier, and ultrasonic transducer to convert high-frequency electrical signals into longitudinal mechanical vibrations, which are transmitted to the micro-tool (MT). The vibrating tool then interacts with the copper wire to induce localized deformation and surface modification. Precise positioning is achieved through a three-dimensional stage driven by a stepper motor and a TB6600 driver (Pufeide Co., Ltd., Wenzhou, China), while one STM32-based controller, together with an oscilloscope, host computer, and optical microscope, enables real-time signal regulation, process monitoring, and data acquisition, thereby forming a coordinated environment for ultrasonic-assisted micro-machining.

### 2.2. Working Mechanism of Ultrasonic Actuation Module

**Figure 2:**
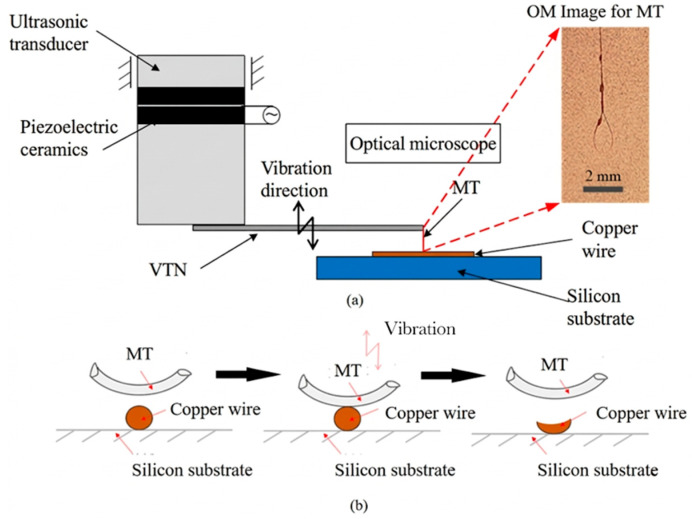
Schematic diagram of the ultrasonic processing module: (**a**) structure; (**b**) ultrasonic machining process.

The ultrasonic actuation module is based on a piezoelectric transducer operating at a working frequency of 40 kHz, as shown in Figure 2a. The transducer is driven by a programmable signal generator and a power amplifier, allowing independent control of excitation frequency and voltage amplitude. The generated sinusoidal signal is amplified and applied to the transducer, producing forced vibration through the vibration transmission needle (VTN) at the tool tip. An oscilloscope is used to monitor the applied voltage and current in real time to ensure stable excitation conditions. A three-dimensional positioning stage provides precise movement of the workpiece, allowing the micro-tool (MT) to act on the workpiece and predetermined positions and along specified trajectories, enabling a stable and controllable processing process. Figure 2b shows that, under ultrasonic excitation, the induced high-frequency oscillation modifies the contact and pressure conditions between the MT and the copper wire, thereby enabling controllable micro-forming of the wire while facilitating real-time observation of deformation evolution.

**Figure 3:**
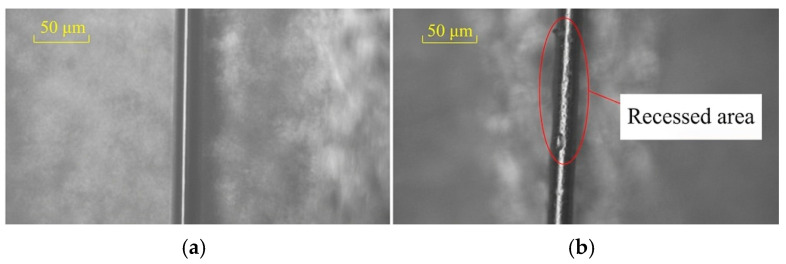
Optical observation of a fixed copper wire: (**a**) CuMW in the initial state; (**b**) CuMW after ultrasonic processing at 38.9 kHz.

The principle of fine copper wire processing is to concentrate the high-frequency vibration energy generated by the ultrasonic transducer, focusing it on the copper wire to achieve high-frequency vibration rolling, thereby accomplishing post-processing of the copper wire. Fixing fine wire materials has always been a challenge in their processing. In this method, a layer of welding glue is applied to the surface of a 2 mm × 2 mm rectangular neodymium magnet, and the copper wire is stretched by gravity at both ends, ensuring it fully adheres to the surface of the rectangular neodymium magnet. The use of the rectangular neodymium magnet facilitates transfer to a metallographic microscope for observation and fixation on the processing platform. The combination workpiece is fixed to the platform through the strong magnetism of the neodymium magnet, creating a strong suction force that secures it onto the platform. The initial state of a fixed copper wire is shown in Figure 3a. After ultrasonic processing, a recessed area can be observed on the fixed copper wire, as shown in Figure 3b.

**Figure 4:**
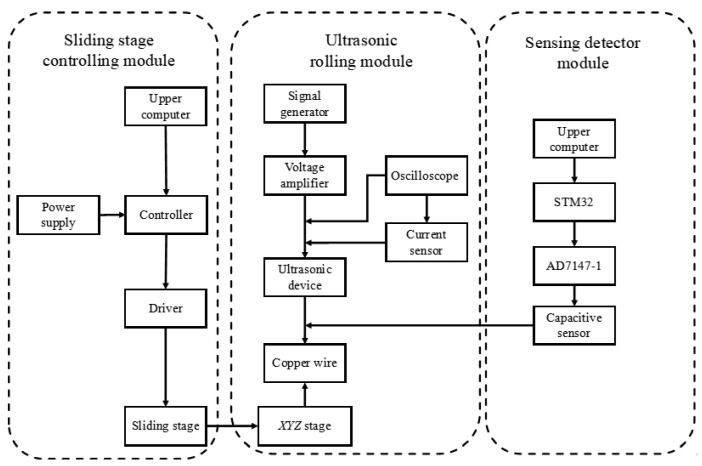
The work flow of the ultrasonic machining platform.

The slide control device consists of a slide, a stepper motor, a stepper motor driver, a controller, and a host computer, as shown in the schematic diagram in Figure 4. The host computer runs a pre-written program and sends control commands to the controller. After processing, the controller outputs pulse signals to the driver, thereby driving the stepper motor to rotate in the set direction and angle, achieving precise motion control of the slide.

## 3. Characteristics of Ultrasonic Forming on Fine Copper Wires

### 3.1. Ultrasonic Energy Transmission and Contact Stiffness

During ultrasonic processing, the vibration energy generated by the transducer is transmitted through the vibration transfer rod to the MT and finally concentrated at the contact region between the MT and the copper wire. The efficiency of ultrasonic energy transmission strongly depends on the contact stiffness of the MT system. When flexible support materials like rubber or silicone are used, part of the ultrasonic vibration energy is absorbed or dissipated due to elastic deformation of the supports, resulting in reduced vibration amplitude at the processing interface. Consequently, the effective stress acting on the copper wire is insufficient to induce stable plastic deformation.

**Figure 5:**
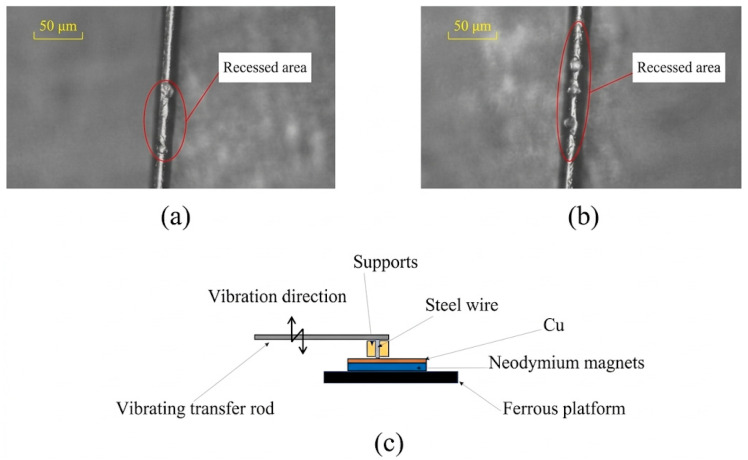
Schematic diagram of the slide module: (**a**) copper wire-machined depression morphology; (**b**) copper wire-machined forming morphology; (**c**) schematic diagram of ultrasonic processing.

In contrast, rigid support structures, such as hard plastic rings, provide higher contact stiffness and reduced energy dissipation. Figure 5 illustrates the schematic of the ultrasonic processing module and the resulting machined morphologies, which further confirm that increased support stiffness significantly improves forming stability and effectiveness.

Table 1 shows an obvious rigidity gradient of the experimental materials. The core processing logic of this study lies in achieving effective forming of thin copper wires. The MT must have a higher elastic modulus to ensure that the ultrasonic vibration energy is highly focused at the contact interface. In this process, the selection of the support material profoundly affects the forming quality through the “base effect”, which refers to the capability of the supporting substrate to respond to ultrasonic pressure. Compared with soft plastics (<1.5 GPa) or rubbers (<0.1 GPa), which tend to produce “soft shrinkage” deformation and dissipate energy, hard plastic rings (>2.0 GPa) provide stable and rigid support due to their higher elastic modulus. This enhanced “base effect” significantly reduces the ineffective loss of ultrasonic energy at the support end, improves the effective amplitude at the processing interface, and thereby achieves an excellent processing depth of 0.95 μm at a low feed rate of 1 mm/min.

A dual-shaft ball slide with an effective travel of 200 mm was used. Due to the size constraints of the slide stage structure, the compatible stepper motor models are limited to types 42 and 57. For stepper motors, the larger the frame size, the greater the holding torque and operating torque, and the stronger the load capacity, although the operating speed will correspondingly decrease. Considering that the experiment requires a relatively low slide speed but a higher load capacity, a type 57 stepper motor was ultimately selected.

The driver uses a TB6600 stepper motor driver (Pufeide Co., Ltd., Wenzhou, China), which is a two-phase hybrid stepper motor driver, suitable for two-phase stepper motors with a phase current not exceeding 4.0 A. It can meet the driving needs of small- and medium-sized automation equipment and experimental setups. The 57-type stepper motor (Leadshine, Shenzhen, China), TB6600 stepper motor driver, PC66 programmable automation controller (Omron, Kyoto, Japan), and 24 V DC power supply are electrically connected, and the driver pulse count is set to 6400 pulses/revolution.

**Figure 6:**
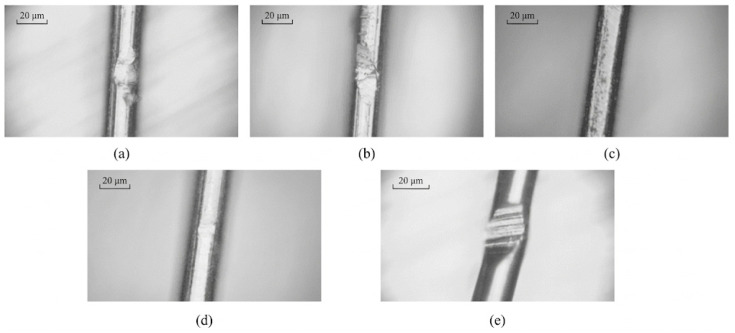
Images of the surface morphology of copper wires subjected to different conditions: (**a**) rubber ring; (**b**) solid silica gel; (**c**) hollow silica gel; (**d**) soft plastic ring; (**e**) hard plastic ring.

Figure 6a–e present microscopic images of the surface morphology of copper wires subjected to different conditions, each with a scale bar of 20 μm. As shown, the surface characteristics vary significantly depending on the treatment applied. In Figure 6a,b, localized surface damage and adhered deposits are evident, appearing as irregular protrusions or peeling regions. The surface in Figure 6c appears relatively uniform, although a certain degree of roughness is still observable. In Figure 6d, the surface morphology is comparatively smooth, with a noticeable reduction in defects. In contrast, Figure 6e exhibits more regular transverse textures. Overall, these observations indicate that different processing or experimental conditions markedly influence the microscopic surface morphology of the steel wire, resulting in variations in surface roughness, defect features, and their distribution.

**Figure 7:**
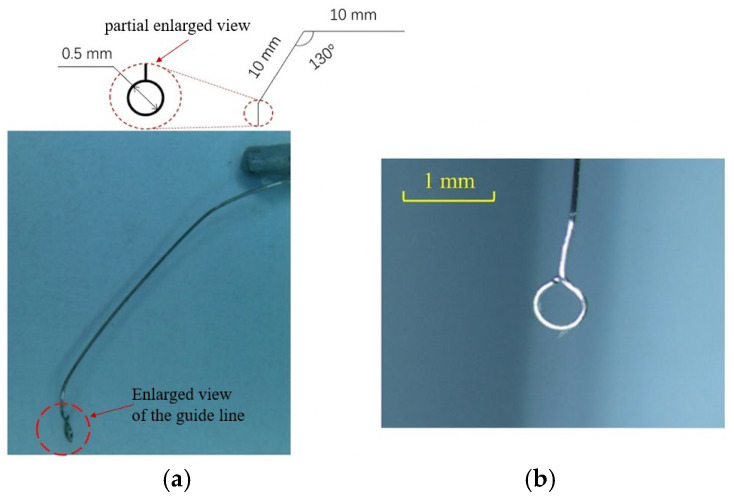
Photograph of the proposed micro-tool structure: (**a**) copper wire-assisted machining head fabrication; (**b**) rigid 304 steel MT.

The diagram in Figure 7 illustrates a simplified mechanical structure. On the left, there is a small circular component connected to a thin vertical rod at the top, with a marked dimension of 0.5 mm. On the right, a bent structure is formed by two line segments, each 10 mm in length, creating an included angle of about 130°, with a horizontal segment at the upper end. The two red dashed circles highlight key locations, and the dashed lines between them indicate the relationship between the two parts.

The MT is mounted at the end of the ultrasonic tool and functions as the direct interface between the vibrating actuator and the copper wire. The tool tip is fabricated with a characteristic dimension comparable to the wire diameter, ensuring localized contact during processing.

During operation, the MT undergoes high-frequency axial vibration with a peak-to-peak displacement on the order of micrometers. The ultrasonic vibration induces intermittent contact between the tool and the wire, which effectively modifies the local stress state and facilitates material deformation.

Figure 7 also shows a photograph of the proposed micro-tool structure. The left image presents the overall configuration, a thin, elongated metal wire extends in a gentle curve, forming a small structure at its tip, which is highlighted by a red dashed circle. The right image provides a magnified view of this terminal structure, clearly revealing that the wire is bent into an approximately circular loop, connected to a straight segment at the top.

The copper wire is fixed on a precision positioning stage that provides controlled translational motion with micrometer-level resolution. The wire is mechanically constrained to prevent rigid-body motion while allowing local deformation at the contact region. The relative position between the MT and the wire is adjusted prior to ultrasonic excitation to ensure repeatable processing conditions.

The system operation is controlled through a synchronized control unit that coordinates ultrasonic excitation and mechanical positioning. During processing, the excitation signal is first applied to the transducer to establish stable vibration. The MT is then brought into contact with the copper wire under controlled conditions. The processing duration and excitation parameters are adjusted according to the desired level of deformation or surface modification. After processing, the ultrasonic excitation is switched off before retracting the MT.

**Figure 8:**
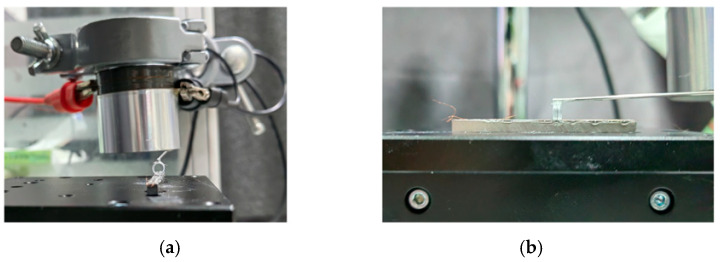
Schematic diagram of the ring-shaped MT support assembly: (**a**) front view; (**b**) side view.

Figure 8a illustrates a precision experimental setup used for materials processing and testing. In Figure 8b, a different perspective reveals a delicate specimen placed on a flat substrate.

### 3.2. Sensing Module of the Ultrasonic Micro-Processing System

**Figure 9:**
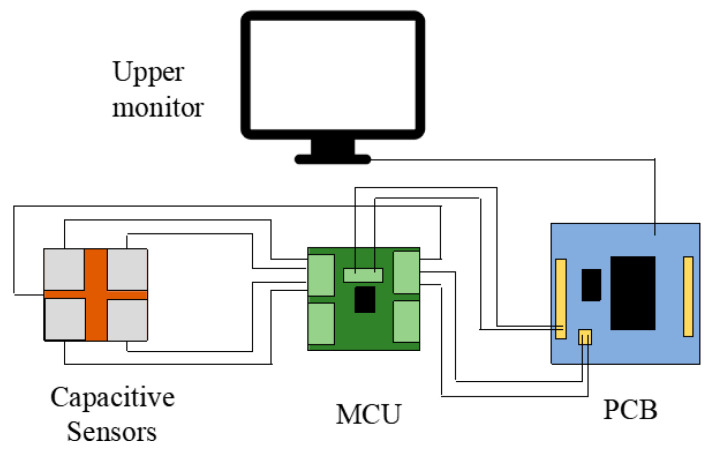
Schematic diagram of the control and sensing architecture of the ultrasonic micro-processing system.

Figure 9 shows the sensing architecture of the ultrasonic micro-processing system. The capacitive sensors acquire pressure-related signals and transmit them to the MCU for signal processing. The processed data are then communicated to the PCB and displayed on the upper monitor for real-time monitoring and analysis.

**Figure 10:**
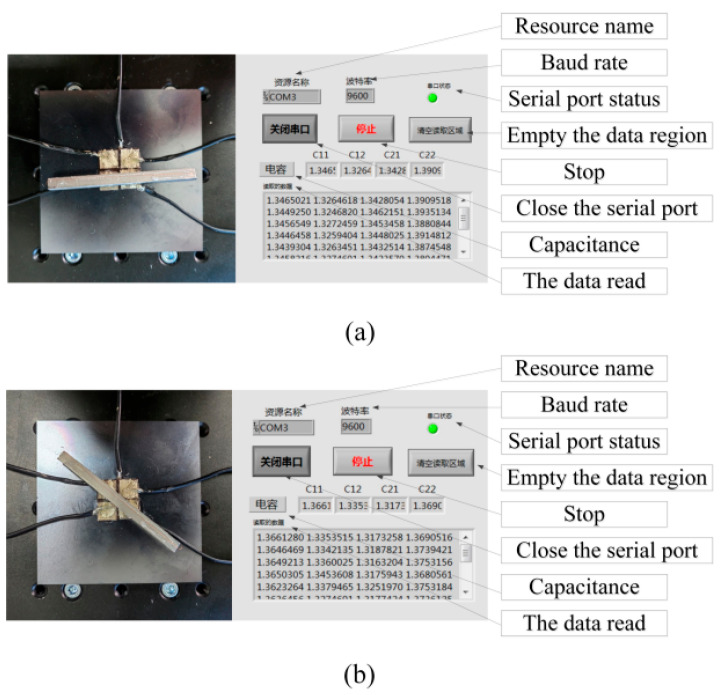
Position-related sensing data acquisition interface under two different states: (**a**) interface at positions C21, C22; (**b**) interface at positions C11, C22.

In Figure 10a, the metal beam is positioned parallel to the electrode structure and connected to the host computer via a serial port. The interface displays the resource name, baud rate, serial port status, and real-time capacitance readings. In Figure 10b, the metal beam is repositioned, leading to noticeable changes in the measured capacitance values shown on the interface. By comparing the capacitance data in these two conditions, real-time monitoring and analysis of structural orientation of displacement can be achieved, highlighting the system’s sensitivity and stability in detecting subtle deformations and acquiring electrical signals.

The MT material and feed speed significantly influence forming performance. Low stiffness MT tends to deflect under ultrasonic excitation, leading to unstable contact and dispersed deformation, whereas MT with higher stiffness provides better structural stability and consistent plastic deformation. The ring-shaped design further concentrates stress and enhances distributed energy. Low moving speed can secure contact time and forming depth.

Overall, the forming mechanism of fine copper wires under ultrasonic excitation can be attributed to efficient vibration energy transmission, high contact stiffness, stable micro-tool geometry, and sufficient energy accumulation controlled by feed speed. These factors collectively determine the forming stability and achievable deformation depth.

## 4. Results and Discussion

The ultrasonic forming behavior of fine copper wires observed in this study is the result of the combined effects of ultrasonic vibration transmission, processing head structural stability, support stiffness, and feed speed. The experimental results provide clear evidence of how these factors jointly influence forming stability and deformation characteristics.

The principle of micro copper wire processing proposed in this paper is to effectively concentrate the high-frequency vibration energy generated by the ultrasonic transducer, allowing the vibration focus to act on the surface of the copper wire, and achieve post-processing of the copper wire through high-frequency vibration rolling. The fixation of fine wires during the processing has always been one of the key difficulties. To address this, this paper uses a strip-shaped neodymium magnet with a cross-section of 2 mm × 2 mm, evenly coated with a layer of welding glue on its surface, and applies gravitational stretch to both ends of the copper wire, so that it attaches fully and is fixed to the surface of the magnet.

**Figure 11:**
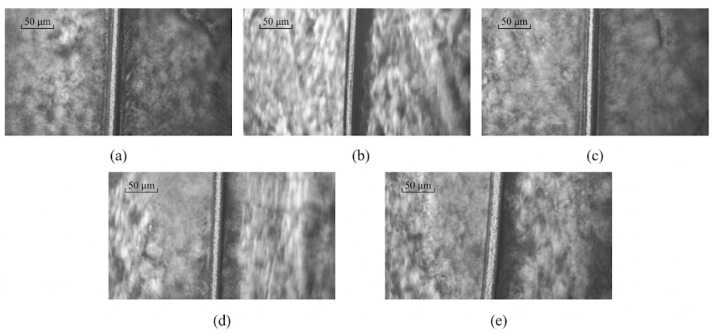
Machining effects of copper wire at different feed speeds: (**a**) 20 mm/min; (**b**) 15 mm/min; (**c**) 10 mm/min; (**d**) 5 mm/min; (**e**) 1 mm/min.

**Figure 12:**
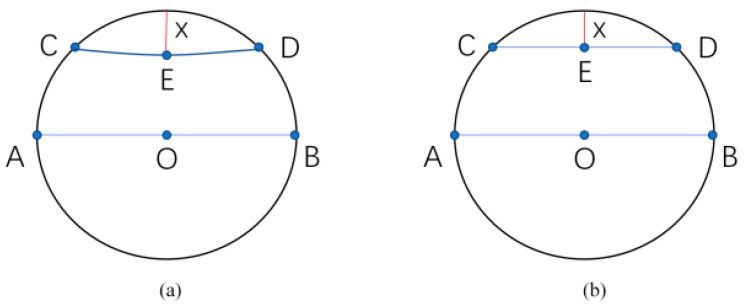
The relationship between the machining depth *X* and the cross-section of copper wire: (**a**) theoretical machining cross-section; (**b**) calculated machining cross-section (Note: *O* denotes the center of the cross-section of the copper wire; *AB* is the diameter passing through the center; *CD* represents the chord formed by indentation of the micro-tool; *E* is the midpoint of chord *CD*; *X* indicates the machining depth from the midpoint to the arc).

**Figure 13:**
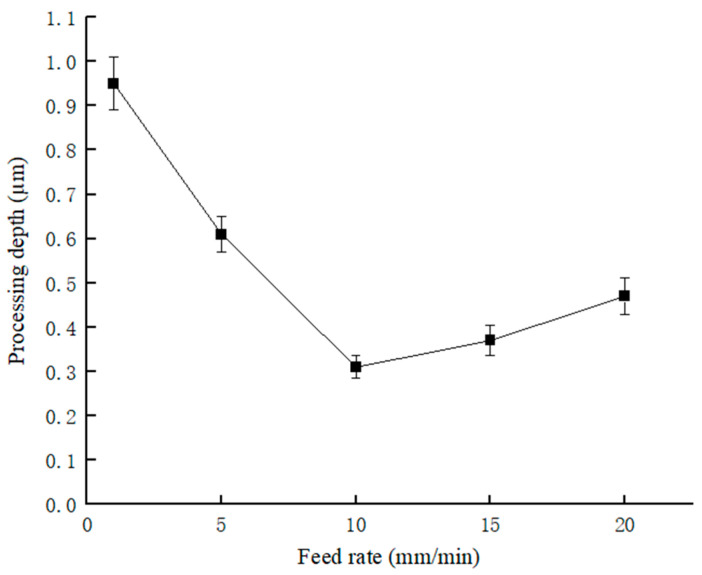
The relationship between feed rate and the measured depth of machining.

Figure 11a–e are optical microscopic images of micrometer-scale copper wires at a scale of 50 μm, presenting the machining effects under different feed velocities. A vertical copper wire is clearly visible in the center of each image with a distinct outline. The images intuitively reflect the variation in the surface forming traces and recessed depth of the copper wire with the feed speed. The forming traces become progressively clearer and the recessed depth increases as the feed speed decreases. This is a direct morphological manifestation of the feed speed effect on the ultrasonic micro-forming of copper wires, which is consistent with the quantitative variation trend of machining depth with feed speed shown in Figure 12 and Figure 13.

At the microscale, this vibration-assisted mechanism promotes intermittent contact and stress redistribution, which distinguishes the present process from conventional quasi-static micro-processing. Figure 12 illustrates the relationship between a circle representing the cross-section of copper wire and the machining depth *X*. The center of the circle is *O*, and *AB* is the diameter passing through the center. *CD* at the top is a chord of the circle, E is the midpoint of chord *CD*, and *EX* represents the perpendicular distance from the midpoint of the chord to the arc. The forming shape *CED* is closely related to the shape of the MT.

According to the geometric model for the cross-section of the micro copper wire shown in Figure 12, the plastic deformation cross-sectional area A(X) generated at a machining depth X follows the analytical formula below:

(1) $$A\left(X\right)\;=\;R^{2}\arccos\left(\frac{R-X}{R}\right)-(R-X)\sqrt{2\mathrm{RX}-X^{2}}$$

where R is the initial radius of the copper wire (0.05 mm). This formula precisely defines the mapping relationship between the geometric dimensions of the deformation zone and the machining depth. Under microscale machining conditions (X ≪ R), the above analytical expression can be simplified as

(2) $$A\;\approx \;\frac{2}{3}\cdot W\cdot X$$

Here, “W” denotes the processing width measured from the SEM images.

In Figure 13, the experimental relationship between feed rate and average depth of machining is illustrated. The horizontal axis represents the feed rate (mm/min), and the vertical axis represents the processing depth. The measured results show that the average depth of machining is greatest at a feed rate of 1 mm/min, reaching an average 0.95 μm. It is the result of the combined effect of the ultrasonic energy density distribution and the contact action mechanism between the MT and the workpiece. Under the conditions of approximately the same material displacement V and constant tool oscillation energy E, when the feed rate increases from 1 mm/min to 10 mm/min, the ultrasonic energy density per unit length of the copper wire decreases with the increase in feed rate, which directly leads to a significant reduction in the processing depth. The average machining depth reaches the maximum value at 1 mm/min, and decreases to 0.61 μm and 0.31 μm at 5 mm/min and 10 mm/min, respectively. However, when the feed rate exceeds 10 mm/min, the processing depth shows a slight upward trend. At 15 mm/min and 20 mm/min, the average depths reach 0.37 μm and 0.47 μm, respectively. This is because at high feed rates, the intermittent contact force increases slightly. Overall, the average depth of machining exhibits a trend of first decreasing and then slightly increasing with increasing feed rate.

**Figure 14:**
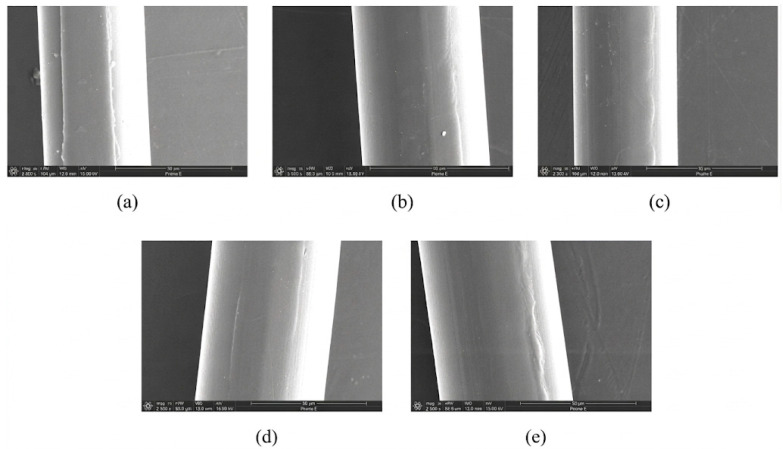
SEM longitudinal cross-sectional morphologies of the machined region of fine copper wires at different feed rates: (**a**) 1 mm/min; (**b**) 5 mm/min; (**c**) 10 mm/min; (**d**) 15 mm/min; (**e**) 20 mm/min.

To intuitively evaluate the effects of different process parameters on the forming quality of fine copper wires, the longitudinal section morphology of the machined samples was observed using scanning electron microscopy (SEM). Figure 14 shows the micro-indentation morphology formed on the surface of fine copper wires at feed speeds ranging from 1 mm/min to 20 mm/min. It can be observed from Figure 14 that at a low feed speed of 1 mm/min, the material undergoes the most significant plastic flow due to the most frequent ultrasonic impacts per unit length, forming deep and wide continuous machining grooves with an average depth of 0.95 μm. As the feed speed increases to 5 mm/min and 10 mm/min, the indentation depth shows a remarkable downward trend, which is attributed to the reduction in single-point energy accumulation. However, when the speed is further increased to 15 mm/min and 20 mm/min, although the overlap ratio of single impacts decreases, the cross-sectional morphology exhibits better geometric consistency, and the depth rises slightly at 20 mm/min. This morphological evolution reflects the competition mechanism of ultrasonic energy under different feeding efficiencies. To further quantitatively reveal this mechanism, the standard processing parameters, namely the reduction ratio ε and the energy conversion coefficient *k*, are introduced in the subsequent sections.

To further reveal the forming mechanism of microscale copper wires under ultrasonic excitation and enhance the universality of the research findings, the standard processing parameters (i.e., reduction ratio ε and energy conversion coefficient *k*) are introduced to quantitatively characterize the forming process in combination with the longitudinal cross-sectional morphology and the energy conversion model.

The reduction ratio is defined as the percentage of the machining depth *X* to the initial diameter *D* of the copper wire:

(3) $$\varepsilon =\frac{X}{D}$$

Combined with the experimental results in Figure 13 and the SEM observations of longitudinal morphologies, the characterization results at different feed rates are presented in the following Table 2.

The experimental results indicate a discrepancy between the measured processing width *W* and the theoretical chord length derived from the ideal circular segment model. This phenomenon is primarily attributed to the elastic recovery of the copper wire and the localized energy focusing characteristics of the micro-tool tip under high-frequency vibration. In the subsequent energy efficiency analysis, the effective deformation cross-sectional area *A* was calculated based on SEM observations.

Subsequently, the energy conversion efficiency coefficient k is introduced to evaluate the effective utilization rate of ultrasonic energy in plastic deformation, and the formula for calculating k is given as follows:

(4) $$k=\frac{\sigma \cdot V}{E}$$

where σ is the yield strength of the micro copper wire (a typical value of *σ* ≈ 200 MPa, i.e., 2 × 10^8^ N/m^2^, is adopted for cold-worked hardened fine copper wires); *V* is the displacement (migration) volume of the material within a single vibration cycle; E is the ultrasonic input energy of the tool within a single vibration cycle. According to the monitoring data of the experimental platform, the working voltage of the system is *U* = 58 V, the current is *I* = 950 mA (0.95 A), and the phase difference between voltage and current is *ϕ* = $68.6^{{}^{\circ}}$. The active electric power P of the system is given by

(5) $$\begin{array}{c} P=U\cdot I\cdot cos\phi =58\times 0.95\times \cos\left(68.6^{{}^{\circ}}\right)\approx 20.1W \end{array}$$

Given the ultrasonic operating frequency *f* = 38.9 kHz, the ultrasonic input energy within a single vibration cycle is

(6) $$\begin{array}{c} E=\frac{P}{f}=\frac{20.1}{38,900}\approx 5.17\times 10^{-4}\;J \end{array}$$

It should be noted that Equation (5) calculates the active electrical power input to the system, rather than the effective mechanical power directly acting at the tool tip. Consequently, the input energy *E* is derived from the overall electrical power. Therefore, the subsequently obtained efficiency *k* serves as a relative efficiency indicator for comparative analysis under different parameters, rather than representing the absolute energy conversion efficiency at the tool–workpiece interface. The displacement volume *V* during a single vibration is not only restricted by the cross-sectional deformation area *A* enclosed by chord *CD* and the circular arc in Figure 12, but also strictly depends on the feed rate *v*. Given the ultrasonic operating frequency f, the longitudinal feed step of the tool within a single vibration cycle is *v*/*f*. Therefore, the displacement volume generated in a single vibration can be approximately expressed as

(7) $$\begin{array}{c} V=A\cdot \lambda =A\cdot \frac{v}{f} \end{array}$$

Substituting the expressions for *v* and *E* into the defining equation of *k*, we obtain

(8) $$\begin{array}{c} k=\frac{\sigma \cdot V}{E}=\frac{\sigma \cdot A\cdot \frac{v}{f}}{\frac{P}{f}}=\frac{\sigma \cdot A\cdot v}{P} \end{array}$$

Substituting each group of data into the formula, the efficiency results at different feed rates are obtained as follows.

Based on the above formulaic calculations and analyses, this indicates a tendency that the following important conclusions can be drawn. As presented in Table 3, at a feed rate of 1 mm/min, the machining depth and reduction ratio reach their maximum values, whereas the energy conversion efficiency k is the lowest. This indicates that at low feed rates, the micro-tool delivers tens of thousands of high-frequency repeated impacts at nearly the same position, causing most of the ultrasonic energy to be dissipated in the elastic recovery of the material and frictional heat generation at the contact interface. As the feed rate increases to 20 mm/min, the single-point forming depth decreases slightly, but the k value rises by nearly one order of magnitude. This suggests that a higher feed rate helps distribute ultrasonic vibration energy more uniformly along the longitudinal path, reduces ineffective repeated indentation, and thus improves the contribution of unit energy to material volume migration. The experimental results reveal the restrictive relationship between machining depth and energy efficiency: a lower feed rate should be adopted to ensure the reduction ratio for high-precision requirements, while a properly increased feed rate can achieve better energy utilization for efficiency-prioritized machining tasks.

From a functional perspective, the proposed setup can be regarded as an ultrasonic microactuation system for microscale metal processing. Although the actuation module itself operates at the mesoscale, the induced mechanical interaction and material response occur at the microscale. The choice of a strip-shaped neodymium magnet not only facilitates the transfer of samples to a metallographic microscope for observation but also makes it easy to achieve prompt and stable fixation with the processing platform. During processing, the strong magnetism of the neodymium magnet ensures reliable adhesion between the combined workpiece and the platform, thereby guaranteeing processing stability.

In order to achieve effective processing of copper wire, the material of the processing head needs to have a certain hardness and wear resistance, with its stiffness and elastic modulus higher than that of copper. At the same time, to effectively focus vibrational energy, the contact area of the processing head should be as small as possible. Among materials that meet these conditions, both fiberglass and steel wire have good applicability. In addition, the structure of the processing head should facilitate contact with the workpiece and avoid energy dispersion.

Compared with other ring-shaped structures such as square or polygonal ones, a circular ring-shaped head has advantages like simple processing techniques, lower cost, and smaller contact area. Therefore, this study uses a circular ring-shaped MT, and its processing procedure is shown in Figure 7. The machining performance of fiberglass and steel wire MT is analyzed, and three machining schemes are designed, from which the optimal scheme is selected for continuous processing tests. An ultrasonic transducer with a natural frequency of 40 kHz and a rated power of 60 W is used, operating at 38.9 kHz. Oscilloscope measurements show an operating voltage of 58 V and a current of 950 mA, with a voltage/current phase difference of 68.6°.

Since 304 steel does not have the same resilience as fiberglass, it is prone to deformation under external forces affecting processing accuracy. To ensure the dimensional stability of 304 steel rings during processing, adding internal supports to the rings is particularly important. When the ring is subjected to external forces during processing, the internal supports can provide a counteracting force to offset some of the external force, helping the ring maintain its original shape, thereby ensuring consistent processing outcomes and improving processing precision and quality.

Compared to other ultrasonic micro-machining methods, we have developed a micro-machining tool with a flexible ring structure to improve the controllability and stability of processing micron-scale copper wires. Traditional ultrasonic micro-machining typically relies on rigid tool heads for energy transmission, which can lead to uneven contact, local stress concentration, and instability in vibration coupling when processing slender, flexible micron-scale copper wires, thereby affecting machining accuracy and consistency. To address these limitations, the proposed flexible ring structure introduces adjustable compliance units, giving the tool head a certain degree of adaptive capability in the radial direction, allowing it to cover and contact micron-scale copper wires more uniformly. Under ultrasonic vibration, this structure can achieve multi-point collaborative loading, reducing stress concentration from single-point contact while improving the uniformity of vibration energy distribution on the workpiece surface. In addition, the flexible ring structure can to some extent buffer dynamic impacts during the machining process, reducing dimensional deviations caused by amplitude fluctuations.

In this study, the effects of rubber rings, solid silicone rods, hollow silicone rings, soft plastic rings, and hard plastic rings as support elements were tested separately. When using rubber rings as supports, the elasticity of the rubber can absorb part of the external force and return to its original shape after the force is removed, effectively maintaining the shape of the ring. When using hard plastic rings, their stronger support can more effectively prevent deformation of the ring. Different support types were evaluated for their ability to maintain the ring shape and enhance the processing performance.

As shown in Figure 6 and Figure 11, the forming effects obtained using different support materials exhibit significant variation. As shown in Figure 6a–d, when flexible supports, such as rubber rings, hollow silicone rings, and solid silicone rods, were employed, the forming depth was shallow and the deformation features were unclear. This is mainly due to their low stiffness, which absorbs and dissipates much of the ultrasonic vibration energy during processing. As a result, the effective vibration amplitude transmitted to the contact interface between the processing head and the copper wire is reduced, leading to insufficient stress for stable plastic deformation.

In contrast, when a hard plastic ring was used as the support (Figure 6e and Figure 11e), a significantly clearer and deeper forming trace was observed. The higher stiffness of the hard plastic ring increases the contact stiffness of the processing system, suppresses energy dissipation, and promotes efficient ultrasonic energy concentration at the processing interface. This confirms that support stiffness plays a critical role in ultrasonic energy transmission and forming effectiveness for fine copper wires.

## 5. Conclusions

An experimental platform for the post-processing of micrometer-scale copper wires based on an ultrasonic MT was proposed in this study, and the influence parameters of the three key factors of MT material, support structure type and feed speed on the ultrasonic micro-forming performance of copper wires were systematically investigated. Feed speed exerted a significant quantitative effect on the average machining depth and surface forming quality of the copper wires. The average depth reached a maximum of 0.95 μm at a feed speed of 1 mm/min, and decreased to 0.61 μm and 0.31 μm in turn as the feed speed increased to 5 mm/min and 10 mm/min. When the feed speed exceeded 10 mm/min, the average machining depth showed a slight rebound, reaching 0.37 μm and 0.47 μm at 15 mm/min and 20 mm/min, respectively. Compared with glass fiber MT, the rigid ring-shaped MT made of 304 stainless steel had higher structural stability and could effectively reduce the energy loss of ultrasonic vibration transmission.

At the same time, this study still has certain limitations. The setting of experimental variables is relatively limited, as only the influences of several typical feed speeds and support structures were investigated, without considering the coupling effects of parameters such as ultrasonic vibration frequency and machining pressure. Nevertheless, these findings provide critical guidance and a reliable experimental basis for optimizing ultrasonic micro-forming processes for fine metallic wires.

## Figures and Tables

**Table 1:** Materials used in the experiment and their reference elastic modulus values.

Material Name	Material Application	Elastic Modulus (GPa)
304 Stainless Steel	Micro-tool (MT) Material	193
Glass Fiber	Micro-tool (MT) Material	70–89
Hard Plastic	MT Support	2–10
Soft Plastic	MT Support	0.2–1.5
Rubber/Silicone	MT Support	0.001–0.1
Copper	Workpiece	110–130

**Table 2:** Processing depth, width and reduction ratio of fine copper wires at different feed rates.

Feed Rate *v* (mm/min)	Processing Depth *X* (μm)	Processing Width *W* (μm)	Reduction Ratio *ε* (%)
1	0.95	14.2	0.95
5	0.61	12.5	0.61
10	0.31	11.8	0.31
15	0.37	11.2	0.37
20	0.47	10.8	0.47

**Table 3:** Single vibration displacement volume and energy conversion efficiency of fine copper wires at different feed rates.

Feed Rate *v* (mm/min)	Cross-Sectional Area *A* (μm^2^)	Single Vibration Displacement Volume *V* (μm^3^)	Energy Conversion Efficiency *k* (×10^−9^)
1	8.99	0.00385	1.49
5	5.08	0.01088	4.21
10	2.44	0.01045	4.04
15	2.76	0.01774	6.87
20	3.38	0.02896	11.21

## Data Availability

All test data mentioned in this paper will be made available upon request from the corresponding author’s email with appropriate justification.
